# Prevalence of Virulence Genes Associated with Diarrheagenic Pathotypes of* Escherichia coli* Isolates from Water, Sediment, Fish, and Crab in Aby Lagoon, Côte d'Ivoire

**DOI:** 10.1155/2017/9532170

**Published:** 2017-06-06

**Authors:** Ollo Kambire, Ama Antoinette Adingra, Konan Mathurin Yao, Rose Koffi-Nevry

**Affiliations:** ^1^Department of Biochemistry and Food Sciences, University of Peleforo Gon Coulibaly, BP 1328, Korhogo, Côte d'Ivoire; ^2^Laboratory of Microbiology, Oceanographic Research Center, BP V 18, Abidjan, Côte d'Ivoire; ^3^Laboratory of Biotechnology and Food Microbiology (UFR/STA), University of Nangui Abrogoua, 02 BP 801 Abidjan, Côte d'Ivoire

## Abstract

This study was conducted to characterize virulence genes of* Escherichia coli *isolates from water, sediment, fish, and crab in Aby Lagoon. Serogrouping was performed by EPEC antisera in 113* E. coli *strains. The presence of diarrhea-associated genes* (eae, stx, AggR, elt, *and* est)* was assessed by multiplex PCR using specific primers. Based on the multiplex PCR, sixty-two isolates (42 from water, 19 from sediment, and 1 from crab) were positive for virulence genes, including 34 positive for* elt *(ETEC), 46 positive for* est *(ETEC), 24 positive for both* elt* and* est, *6 positive for* stx *(EHEC), 1 positive for both* stx* +* est*, and 1 positive for both* stx *+* elt.* Genes* eae* (EPEC) and* AggR* (EAEC) were not detected. Nine serogroups (O114, O127, O55, O111, O86, O119, O126, O128, and O142) were identified. This study revealed the presence of diarrheagenic and nondiarrheagenic* E. coli *and potential public health risks if fishery products are not appropriately cooked.

## 1. Introduction

Most* Escherichia coli* strains are a normal inhabitant of the intestinal tract of humans and warm-blooded animals. Despite being usually harmless, various* E. coli* strains have acquired genetic determinants (virulence genes) giving them the capacity to cause illness for both humans and animals. Some strains of* E. coli* are now seen as pathogenic species with remarkable versatility in their ability to cause disease in humans and animals [1].* E. coli* is one of the most frequent causes of diarrhea in children in developing countries [2]. According to Grasso et al. [3] and Tumwine et al. [4], infectious pathotypes of* E. coli *are related to the lack of sanitation and personal hygiene but also the consumption of well water, river water, and other contaminated surface waters.

Diarrheagenic* E. coli* (DEC) is classified on the basis of its epidemiological, clinical, and pathogenic characteristics into the following six different pathotypes: enteropathogenic* E. coli* (EPEC), shiga-toxin producing* E. coli* (STEC) or enterohemorrhagic* E. coli* (EHEC), enterotoxigenic* E. coli *(ETEC), enteroinvasive* E. coli* (EIEC), enteroaggregative* E. coli* (EAEC), and diffuse adherent* E. coli* (DAEC) [1]. Each pathotype expresses a unique set of virulence and colonization factors encoded in the chromosome or in episomal structures [5]. The genes encoding these virulence factors are conserved among strains isolated from different continents [6–9].

Among the* E. coli *pathogenic strains, in most developing countries, EPEC, ETEC, and EAEC are the most common cause of infectious diarrhea in young children [10, 11]. Research into EPEC is intense and provides a good virulence model of other* E. coli* infections as well as other pathogenic bacteria [12]. According to the World Health Organization (WHO) in 1987, most EPEC strains belonged to a series of O antigenic groups known as EPEC serogroups which included O26, O55, O86, O111, O114, O119, O125, O126, O127, O128, O142, and O158 [13]. Serogrouping of* E. coli* based on somatic O antigen used for differentiating diarrheagenic* E. coli* is costly and time-consuming and poorly correlates with the presence of virulence factors. So, in the last decade, modern molecular detection methods were reported in the literature for rapid identification of* E. coli* pathotypes including PCR and multiplex PCR.

In spite of increasing evidence that* E. coli* strains originating from human and animal feces contain several virulence genes, only a few studies have investigated the presence of* E. coli* pathotype in environmental waters [14–18]. The presence of* E. coli* strains with virulence genes profiles similar to EHEC, EPEC, and ETEC in environmental waters has already been reported. To the best of our knowledge, no investigation on* E. coli* pathotypes distribution has been carried out on the estuarine water environments of Côte d'Ivoire. Yet, these environments that receive frequently domestic wastewater and mammalian feces provide important fishery resources. The production of fish and shellfish is estimated, respectively, to be 6.000 and 7.000 tons per year in the Aby Lagoon [19]. Contamination of lagoon waters by* E. coli* pathotypes could have a negative impact on fish, crabs, and other animals in this environment. Thus, a potential public health risk exists if these fishery products were contaminated by these pathotypes on the one hand and on the other hand if the hygiene measures are faulty during cooking. According to Rangel et al. [20], exposure to recreational waters has been linked to high numbers (21 out of 31) of reported* E. coli* O157:H7 disease outbreaks in the United States from 1982 to 2002. In addition, direct ingestion or aerosols of contaminated water during spray irrigation and contaminated vegetable could cause infection.

The aim of this study was to use PCR method to detect four pathotypes of* E. coli *(ETEC, EPEC, EAEC, and EHEC) from water, sediment, fish, and crab samples. During the study, both PCR and culture-based methods were used.

## 2. Materials and Methods

### 2.1. Sampling Sites

**Figure 1:**
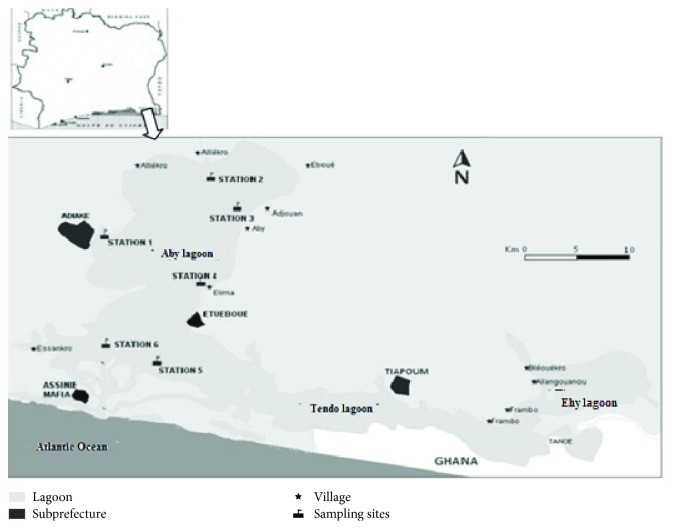
Study area and sampling stations [21].

The Aby Lagoon is located between 2°51 and 3°21 eastern longitude and 5°05 and 5°22 northern latitude southeast. The two main tributaries (*Bia *and* Tanoe*) are escape routes from anthropogenic and mining operations within Aby Lagoon's watershed in Côte d'Ivoire and Ghana (Figure 1). Six sampling stations spread throughout the Aby Lagoon were selected in view of the fact that these stations were subject to various discharges (wastewater, excreta). Station 1 is located near an urban area. Swimming and fishing are practiced here. Station 2 located at the mouth of the river Bia is a fishing zone. Stations 3 and 6, located, respectively, near the latrine on the pile of the Aby and Assomlan villages, are sites where recreational activities are constantly practiced. Stations 4 and 5 are fishing zones.

### 2.2. Sampling

Six campaigns were carried out from June 2010 to March 2011 for the collection of water, sediment, fish, and crab samples. These six campaigns are distributed as follows: two campaigns for the rainy season (June-July), two for the flood season (September-October), and two for the dry season (February-March). At each sampling point, samples of water were collected in sterile glass bottles and those of sediments in stomacher bags. Samples of fish and crabs obtained from fishermen in Aby Lagoon were collected in stomacher bags. A total of 72 water samples and 36 sediment samples were analyzed, consisting of 12 water samples and six sediment samples collected per campaign. Thirty-six fish samples and 36 crab samples were analyzed, with six samples collected per campaign for each. A total of 180 lagoon samples were collected. Collected samples were transported to the laboratory in a cooler containing ice.

### 2.3. Isolation of* Escherichia coli* Strains

A total of 113 strains of* E. coli* were isolated from 72 samples of water, 36 samples of sediment, 36 samples of fish, and 36 samples of crab*. E. coli* isolates from water and sediment were obtained on Eosin Methyl Blue agar (EMB, BIOKAR) through the membrane filtration method. Briefly, 5 mL and 10 mL of water samples were filtered through 0.45 *μ*m cellulose membrane filters (Millipore, Sartorius Stedim Biotech, Germany) and placed on Eosin Methyl Blue agar. For sediment analysis, dilutions (10^−1^, 10^−2^) were first performed with sterile buffer peptone water, and then volumes of 5 mL and 10 mL of each diluted sample were filtered as previously described and placed on Eosin Methyl Blue agar. For fish and crab analysis, 25 g of gut, flesh, and gills of fish and of gut and shell of crab from each sample was added to 225 mL of sterile buffer peptone water contained in a plastic stomacher bag and mixed. Decimal dilutions from this solution were then carried out in buffer peptone water.* E. coli *isolates from fish and crabs were obtained with desoxycholate agar (Becton Dickinson GmbH). All the Petri dishes were incubated at 44.5°C for 24 hours. In addition, isolates were purified on EMB, a selective medium for enterobacteria, and incubated as before. Metallic sheen colonies showing a dark central spot [22] were used as presumptive* E. coli*. Presumptive* E. coli* strains with positive indol, negative citrate, and negative urea were confirmed as* E. coli*.* E. coli* strain of American Type Culture Collection 25922 (ATCC 25922) was used as the control.

### 2.4. Detection of Virulence Genes by PCR

DNA of each isolate was extracted according to the boiling method. Approximately 5 to 10 colonies of an overnight bacterial culture were taken and suspended in 100 *μ*L of distilled water. The mixture was stored at −20°C for 10 min and then boiled at 100°C for 10 min. After centrifugation in a Mikro 220R Hettich centrifuge at 14000 RPM for 10 min, supernatants were used for PCR amplification. The amplification reactions were carried out in a reaction mixture of 25 *μ*L containing 10 *μ*L of Master Mix 1x (5PRIME Hot Master Mix 2.5x Dominique DUTSCHER) (France), 1.4 *μ*M concentration (each) of primers (Table 1), and 5 *μ*L of the DNA template. The PCR amplification was performed using a thermocycler system (Applied Biosystems, 2720 Thermal Cycler, USA). The amplification program included an initial denaturation step at 94°C for 2 min, followed by 30 cycles of denaturation (94°C for 1 min), primer annealing (52°C for 1 min), and extension (65°C for 1 min), with a final extension at 65°C for 10 min. PCR products (10 *μ*L) were resolved by electrophoresis on a 2.5% agarose gel (Promega, USA) at 120 mV for 80 min. Agarose gel was then stained with ethidium bromide (Sigma-Aldrich, USA), and the DNA bands were visualized and photographed under UV illumination (UV UVItec, UK). The buffer in the electrophoresis chamber (PCR SCIE-PLAS, China) and in the agarose gel was 1x Tris-borate-EDTA (89 mM Tris-borate, 2.5 mM EDTA).

### 2.5. Serogrouping of* E. coli* Isolates

Detection of virulence strains among the 113* E. coli* isolates was performed by O serogrouping with 12 antisera (Bio-Rad) by the slide agglutination method according to the manufacturer's instructions. The 12 immune sera tested in this study were O55, O26, O111, O86, O119, O127, O125, O126, O128, O114, O124, and O142.

## 3. Results 

Sixty-two strains (55%) of the 113 strains tested were positive for virulence genes. Pathogenic strains of* E. coli *were more isolated in the sediment with a frequency of 70% of the cases, followed by the strains from water (68%). Virulence strains were least observed with the crabs (9%). No pathogenic strain of* E. coli *was detected in fish samples (Table 2).

The four pathotypes of* E. coli* in this study according to the nature of the samples analyzed are shown in Table 3. Two* E. coli* pathotypes were identified, namely, enterotoxigenic* E. coli* (ETEC) with a percentage of 90% and enterohemorrhagic* E. coli *(EHEC) with a prevalence of 10%. These two pathotypes were observed in the samples of water, sediments, and crab. In water samples, 8% and 60% of the pathogenic strains belonged to EHEC and ETEC, respectively. For sediment samples, 2% of the cases of the virulent strains belonged to EHEC and 29% to ETEC. No strains of enteropathogenic* E. coli* (EPEC) and enteroaggregative* E. coli* (EAEC) were identified. The only pathogenic strain identified in the crab samples belonged to ETEC.

**Figure 2:**
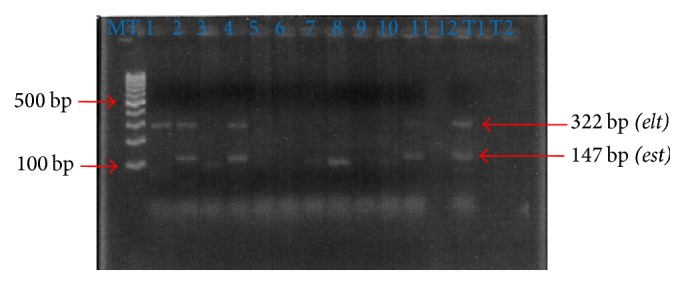
Gel electrophoresis profile of different virulence genes of the potential diarrheagenic* E. coli *isolates. Lane MT: molecular size marker (100 bp DNA ladder). Lane 1:* elt*; lanes 2 and 4:* elt *and* est*; lanes 3, 5, 6, 7, 9, 10, and 12: nonpathogenic* E. coli*; lanes 8 and 11:* est*; T1: positive control* (est, elt)*; T2: negative control.

Table 4 shows the prevalence of virulence genes according to the nature of the samples examined. The genes belonging to ETEC were the most detected with a frequency of 74% and 55% of the cases for the genes* “est”* and* “elt*,*”* respectively. These genes were identified in strains isolated from water, sediment, and crabs with the most important prevalence from the water samples (32% for* “elt”* gene and 50% for* “est”* gene). The ETEC strains harboring* “est”* gene were the most identified (74%). A prevalence of 35% of these strains possessed both the heat-labile toxin gene* (elt)* and the heat-stable toxin gene* (est)*. About 10% of enterohemorrhagic* E. coli *(EHEC) harbored* “stx”* gene. The simultaneous presence of genes* stx *+* est* and* elt *+* stx* was also identified in some strains with a prevalence of 2% for each combination.  Figure 2 shows the PCR amplification products of the target genes studies.

The various serogroups of potential pathogenic* E. coli* according to the nature of samples are shown in Table 5. The results of the serogrouping by antisera showed that 37% of the 62 pathogenic* E. coli *isolates were typeable with the used antisera. Nine serogroups, namely, O114 (14%), O127 (6%), O55 (5%), and 2% for O111, O86, O119, O126, O128, and O142, were identified. The O114 serogroup was the most detected. Different serogroups identified are not specific to each group of pathotype (Table 6).

## 4. Discussion 

Results of the prevalence of potential pathogenic* E. coli *strains found in water (68%) and sediment (70%) samples were similar to those reported by Obi et al. [14] from water and sediment of six rivers in South Africa. These results could be explained by the fact that this lagoon received all effluents. Indeed, several effluents are released often without any treatment in the lagoon. Kambiré et al. [29] showed that the Aby Lagoon was influenced by continental waters. In addition, these authors indicated that most of the household members (93%) living in places without latrines defecated directly into the lagoon. The prevalence of nonpathogenic* E. coli *was 45%. According to Bekal et al. [30],* Escherichia coli* is a normal inhabitant of the intestinal tract of humans and warm-blooded animals. Despite being usually harmless, various* E. coli *strains have acquired genetic determinants (virulence genes) rendering them pathogenic for both humans and animals.

The pathogenic* E. coli* strains found in this study belong to two different pathotypes: ETEC and EHEC. ETEC (90%) represents the most frequent pathotype. This result is similar to those reported by Salem et al. [6]. ETEC was identified as the common cause of infections among tourists visiting Asia, Africa, and South America and also as a common diarrheal pathogen in children in many developing countries of Asia, Africa, and South America [31, 32].

The prevalence of heat-stable toxin gene* (est)* of ETEC was 74% of the strains tested compared to the heat-labile toxin gene* (lt)*, 55%. Other studies showed predominance of* “est”* gene [33, 34]. Several authors have also reported the simultaneous presence of the genes* est* and* lt* in ETEC [32, 35, 36] like in this study. According to Munshi et al. [37], the genes encoding LT (*elt *or* etx*) reside on plasmids that also may contain genes* (est)* encoding ST.

The prevalence of EHEC pathotype was 10%. This frequency is lower than that obtained by Ndlovu et al. [15] which was 15% in their study on the characterization of* E. coli* isolated from surface water sources. However, frequency in this study is higher than that obtained by Obi et al. [14] which was 2% in South African rivers. Our prevalence is approximately similar to those reported by Dadié et al. [38] in 1780 samples of food (meat and dairy products) and 1416 patients in Côte d'Ivoire. One isolate harbored the combination of* stx* and* elt* genes and another* stx *and* est* genes. An association gene was also observed by Moalic and Guennec [39] from* E. coli *strain causing diarrhea in pigs in France. According to Titilawo et al. [16], the lower prevalence of the EHEC pathotype compared to other pathotypes suggests that human fecal contamination is the main source of diarrheagenic* E. coli* pathotypes in the surface water as opposed to contamination from animals. Contrary to the studies of Sidhu et al. [40] and Titilawo et al. [16] in the characterization of* E. coli* from surface water and rivers in Southwestern Nigeria, respectively, genes for EPEC* (eae)* and EAEC* (AggR)* were not detected in this study.

Phenotype assays such as serogrouping with traditional antisera are the routine methods that have been widely used in clinical laboratories [41]. Serogrouping has been shown to be insufficient for the identification of a particular pathotype group. The 12 antisera specific for EPEC group according to the WHO are permitted to detect other pathotype* E. coli *groups like ETEC and EHEC in this study. Nine serogroups were identified in this study. Among the identified serogroups, the O114 serogroup was the most isolated. This serogroup has been the cause of an epidemic of infantile gastroenteritis in England [42].

## 5. Conclusion 

This study shows the presence of pathotypes of* E. coli *in water, sediment, and crab. The pathogenic* E. coli* belongs to two different pathotypes: ETEC and EHEC. ETEC represented the most frequent pathotype. Nonpathogenic strains of* E. coli* were also identified in all samples analyzed, especially in fish samples. Nine serogroups have been identified with O114 as majority group. This study shows the importance of controlling sources of human fecal pollution, such as municipal wastewater management, to reduce potential risks to human health. In this sense, all latrines built on pile should be suppressed. The domestic water must also be treated before being discharged into the lagoon.

## Figures and Tables

**Table 1:** Primers used for PCR in this study [23].

Genes	Sequence (5′ to 3′)	Size (bp)	References
*Eae*	F CCC GAA TTC GGC ACA AGC ATA AGC R CCC GGA TCC GTC TCG CCA GTA TTC G	881	[24]
*Stx*	F GAG CGA AAT AAT TTATAT GTG R TGA TGA TGG CAA TTC AGT AT	518	[25]
*AggR*	F GTA TAC ACA AAA GAA GGA AGC R ACA GAA TCG TCA GCA TCA GC	254	[26]
*Elt*	F TCTCTATGTGCATACGGAGC R CCATACTGATTGCCGCAAT	322	[27]
*Est*	F TTAATAGCACCCGGTACAAGCAGG R CCTGACTCTTCAAAAGAGAAAATTAC	147	[28]

**Table 2:** Distribution of *E. coli* strains.

	Source				Total
	Water	Sediment	Fish	Crab	
Number of strains	62	27	13	11	113
Pathogenic strains	42 (68%)	19 (70%)	0 (0)	1 (9%)	62 (55%)
Nonpathogenic strains	20 (32%)	8 (30%)	13 (100%)	10 (91%)	51 (45%)

**Table 3:** Prevalence of *E. coli* pathotypes.

Source	Frequency (%)				Total
	Water	Sediment	Fish	Crab	
*Pathotype groups*					
EPEC	0	0	0	0	0
EHEC	5 (8%)	1 (2%)	0	0	6 (10%)
EAEC	0	0	0	0	0
ETEC	37 (60%)	18 (29%)	0	1 (2%)	56 (90%)
*Total *	*42 (68%)*	*19 (31%)*	*0*	*1 (2%)*	*62 (100%)*

**Table 4:** Prevalence of virulence genes.

Pathotype groups	Frequency					
	ETEC			EHEC		
Genes	*elt*	*est*	*elt + est*	*stx*	*stx + est*	*stx + elt*
*Source*						
Water	20 (32%)	31 (50%)	12 (19%)	5 (8%)	1 (2%)	1 (2%)
Sediment	13 (21%)	14 (22%)	9 (14%)	1 (2%)	0	0
Crab	1 (2%)	1 (2%)	1 (2%)	0	0	0
*Total *	*34 (55%)*	*46 (74%)*	*24 (35%)*	*6 (10%)*	*1 (2%)*	*1 (2%)*

**Table 5:** Serogroups of potential pathogenic strains typeable.

Serogroups	Water	Sediment	Crabs	Total
O55	2	1	0	3 (5%)
O26	0	0	0	0
O111	1	0	0	1 (2%)
O86	1	0	0	1 (2%)
O119	1	0	0	1 (2%)
O127	3	1	0	4 (6%)
O125	0	0	0	0
O126	1	0	0	1 (2%)
O128	1	0	0	1 (2%)
O114	3	5	1	9 (14%)
O124	0	0	0	0
O142	1	0	0	1 (2%)
*Total N (%)*	*14 (23%)*	*7 (12%)*	*1 (2%)*	*22 (37%)*

**Table 6:** Relationship between virulence genes and O antigens.

Strains	Genes	Serogroups
S1	*est, elt*	ND
S2	*est, elt*	O114
S3	*elt*	ND
E1	*est*	ND
E2	*stx, elt*	O55
C1	*est, elt*	O114
S4	*est, elt*	O114
S5	*est, elt*	O114
E3	*est*	ND
E4	*est, elt*	ND
E5	*est*	O127
S6	*est*	ND
E6	*stx, est*	O114
E7	*est*	O111
S7	*stx*	ND
E8	*elt*	ND
E9	*stx*	ND
E10	*est, elt*	ND
S8	*est, elt*	ND
E11	*est, elt*	O127
S9	*elt*	ND
E12	*elt*	ND
S10	*elt*	ND
E13	*est, elt*	O114
E14	*est, elt*	ND
E15	*est, elt*	ND
E16	*elt*	ND
S11	*est, elt*	O114
E17	*est*	ND
E18	*est*	ND
E41	*est, elt*	ND
E19	*est, elt*	ND
E20	*est*	O114
E21	*stx*	O127
E22	*elt*	ND
E23	*est*	O126
S12	*est*	O127
E24	*est*	ND
E25	*est*	ND
E26	*est*	ND
S13	*est*	ND
E42	*est*	ND
E27	*elt*	ND
E28	*est, elt*	ND
E29	*est*	ND
S14	*est, elt*	O55
E30	*est*	O142
E31	*est*	O128
S15	*est, elt*	ND
E32	*elt*	ND
E33	*stx*	ND
E34	*est*	O119
S16	*est*	O55
S17	*est*	O114
E35	*est, elt*	O86
E36	*est*	ND
E37	*elt*	ND
E38	*est, elt*	ND
E39	*elt*	ND
S18	*est, elt*	ND
E40	*est, elt*	ND
S19	*est*	ND

ND: not determined.

## References

[1] Nataro J. P., Kaper J. B. (1998). Diarrheagenic *Escherichia coli*. *Clinical Microbiology Reviews*.

[2] Mansan-Almeida R., Pereira A. L., Giugliano L. G. (2013). Diffusely adherent Escherichia coli strains isolated from children and adults constitute two different populations. *BMC Microbiology*.

[3] Grasso G. M., Sammarco M. L., Ripabelli G., Fanelli I. (2000). Enumeration of Escherichia coli and coliforms in surface water by multiple tube fermentation and membrane filter methods. *Microbios*.

[4] Tumwine J. K., Thompson J., Katua-Katua M., Mujwajuzi M., Johnstone N., Wood E. (2002). Diarrhoea and effects of different water sources, sanitation and hygiene behaviour in East Africa. *Tropical Medicine and International Health*.

[5] Rúgeles L. C., Bai J., Martínez A. J., Vanegas M. C., Gómez-Duarte O. G. (2010). Molecular characterization of diarrheagenic Escherichia coli strains from stools samples and food products in Colombia. *International Journal of Food Microbiology*.

[6] Salem I. B., Ouardani I., Hassine M., Aouni M. (2011). Bacteriological and physico-chemical assessment of wastewater in different region of Tunisia: Impact on human health. *BMC Research Notes*.

[7] Merchant L. E., Rempel H., Forge T. (2012). Characterization of antibiotic-resistant and potentially pathogenic *Escherichia* colifrom soil fertilized with litter of broiler chickens fed antimicrobial-supplemented diets. *Canadian Journal of Microbiology*.

[8] Cui Y., Li D. F., Yang R. F. (2013). Yang.Shiga toxin-producing *Escherichia coli* O104:H4: an emerging important pathogen in food safety. *Chinese Science Bulletin*.

[9] Oswald E., Schmidt H., Morabito S., Karch H., Marchès O., Caprioli A. (2000). Typing of intimin genes in human and animal enterohemorrhagic and enteropathogenic *Escherichia coli*: characterization of a new intimin variant. *Infection and Immunity*.

[10] Bii C. C., Taguchi T. T., Ouko L. W., Muita N., Kamiya S. (2005). Detection of virulence related genes by multiplex PCR in multidrug-resistance diarrheagenic *E. coli* isolates from Kenya and Japan. *Epidemiology and Infection*.

[11] Wanke C. A. (1995). Enteropathogenic and enteroaggregative strains of Escherichia coli: clinical features of infection, epidemiology, and pathogenesis. *Current Clinical Topics in Infectious Diseases*.

[12] Clarke S. C., Haigh R. D., Freestone P. P., Williams P. H. (2003). Virulence of enteropathogenic *Escherichia coli*, a global pathogen. *Clinical Microbiology Reviews*.

[13] Blanco J. E., Dahbi G., Mora A., Alonso M. P., Varela G. (2006). al..Typing of intimin (eae) genes from enteropathogenic Escherichia coli (EPEC) isolated from children with diarrhea in Montevideo Uruguay: identification of two novel intimin variants (*µ*B and *ξ*R/*β*2B. *Journal of Medical Microbiology*.

[14] Obi C. L., Green E., Bessong P. O., De Villiers B., Hoosen A. A., Igumbor E. O. (2004). Gene encoding virulence markers among Escherichia coli isolates from diarrhoeic stool samples and river sources in rural Venda communities of South Africa. *Water SA*.

[15] Ndlovu T., Le Roux M., Khan W., Khan S. (2015). Co-detection of virulent *Escherichia coli* genes in surface water sources. *PLoS ONE*.

[16] Titilawo Y., Obi L., Okoh A. (2015). Occurrence of virulence gene signatures associated with diarrhoeagenic and non-diarrhoeagenic pathovars of Escherichia coli isolates from some selected rivers in South-Western Nigeria. *BMC Microbiology*.

[17] Hamilton M. J., Hadi A. Z., Griffith J. F., Ishii S., Sadowsky M. J. (2010). Large scale analysis of virulence genes in *Escherichia coli* strains isolated from Avalon Bay, CA. *Water Research*.

[18] Lauber C. L., Glatzer L., Sinsabaugh R. L. (2003). Prevalence of pathogenic *Escherichia col*i in recreational waters. *Journal of Great Lakes Research*.

[19] Sankare Y., Joanny T., Amon-Kothias J. B. (2010). Evaluation des ressources maritimes halieutiques démersales et thonières de la Côte d'Ivoire. *Rapport d'exécution de Convention CRO/PAGDRH*.

[20] Rangel J. M., Sparling P. H., Crowe C., Griffin P. M., Swerdlow D. L. (2005). Epidemiology of Escherichia coli O157:H7 outbreaks, United States, 1982–2002. *Emerging Infectious Diseases*.

[21] Kambire O., Adingra A. A., Eblin S. G., AKA N., Kakou A. C., Koffi N. R. (2014). Caractérisation des eaux d'une lagune estuarienne de la Côte d'Ivoire: la lagune Aby. *Larhyss Journal*.

[22] Nguyen N. H., Tô M. C., Carles M., Tripodi A., Bodin G. (2000). Etude de 91 souches *d'Escherichia coli* responsables de la maladie de l'œdème du porcelet dans le sud du Viet. *Nam. Revue Méd. Vét*.

[23] Toma C., Lu Y., Higa N. (2003). Multiplex PCR assay for identification of human diarrheagenic *Escherichia coli*. *Journal of Clinical Microbiology*.

[24] Oswald E., Schmidt H., Morabito S., Karch H., Marches O., Caprioli A. (2000). Typing of intimin genes in human and animal enterohemorrhagic and enteropathogenic *Escherichia coli*: characterization of a new intimin variant. *Infection and Immunity*.

[25] Yamasaki S., Lin Z., Shirai H. (1996). Typing of verotoxins by DNA colony hybridization with poly- and oligonucleotide probes, a bead-enzyme-linked immunosorbent assay, and polymerase chain reaction. *Microbiology and Immunology*.

[26] Ratchtrachenchai O. A., Subpasu S., Ito K. (1997). Investigation on enteroaggregative Escherichia coliinfection by multiplex PCR. *Bull. Dept. Med. Sci*.

[27] Tamanai-Shacoori Z., Jolivet-Gougeon A., Pommepuy M., Cormier M., Colwell R. R. (1994). Detection of enterotoxigenic *Escherichia coli* in water by polymerase chain reaction amplification and hybridization. *Canadian Journal of Microbiology*.

[28] Hornes E., Wasteson Y., Olsvik O. (1991). Detection of *Escherichia coli* heat-stable enterotoxin genes in pig stool specimens by an immobilized, colorimetric, nested polymerase chain reaction. *Journal of Clinical Microbiology*.

[29] Kambiré O., Adingra A. A., Kakou C. A., Koffi-Nevry R. (2012). Indicateurs de pollution fécale dans une lagune tropicale à forte influence continentale (lagune Aby, Côte d’Ivoire). *Agronomie Africaine*.

[30] Bekal S., Brousseau R., Masson L., Prefontaine G., Fairbrother J., Harel J. (2003). Rapid identification of *Escherichia coli* pathotypes by virulence gene detection with DNA microarrays. *Journal of Clinical Microbiology*.

[31] Qadri F., Svennerholm A. M., Faruque A. S. G., Sack R. B. (2005). Enterotoxigenic Escherichia coliin developing countries: epidemiology, microbiology, clinical features, treatment, and prevention. *Clinical Microbiology Reviews*.

[32] Nessa K. D., Ahmed J., Islam F. L., Kabir, Hossain A. M. (2007). Usefulness of a multiplex PCR for detection of diarrheagenic *Escherichia coli* in a diagnostic microbiology laboratory setting. *Bangladesh J. Med. Microbiol*.

[33] Shaheen H. I., Khalil S. B., Rao M. R. (2004). Phenotypic profiles of enterotoxigenic *Escherichia coli* associated with early childhood diarrhea in rural Egypt. *Journal of Clinical Microbiology*.

[34] Sehand K. A., Layla I. F. S. (2010). Identification of different categories of diarrheagenic Escherichia coli in Stool samples by using Multiplex PCR technique. *Asian Journal of Medical Sciences*.

[35] Aranda K. R. S., Fabbricotti S. H., Fagundes-Neto U., Scaletsky I. C. A. (2007). Single multiplex assay to identify simultaneously enteropathogenic, enteroaggregative, enterotoxigenic, enteroinvasive and Shiga toxin-producing *Escherichia coli* strains in Brazilian children. *FEMS Microbiology Letters*.

[36] Kalnauwakul S., Phengmak M., Kongmuang U., Nakaguchi Y., Nishibuchi M. (2007). Examination of diarrheal stools in Hat Yai City, South Thailand, for *Escherichia coli* O157 and other diarrheagenic *Escherichia coli* using immunomagnetic separation and PCR method. *Southeast Asian Journal of Tropical Medicine and Public Health*.

[37] Munshi S. K., Rahman M. M., Noor R. (2012). Detection of Virulence Potential of Diarrhoeagenic Escherichia coli Isolated from Surface Water of Rivers Surrounding Dhaka City. *Journal of Bangladesh Academy of Sciences*.

[38] Dadié A., Karou T., Adom N., Kétté A., Dosso M. (2000). Isolement d'agents pathogènes entériques en Côte d'Ivoire *Escherichia coli* O157:H7 et *E. coli entéroagrégant*. *Bull. Soc. Pathol. Exot*.

[39] Moalic P. Y., Guennec J. L. (2000). Prévalence des facteurs de virulence de souches d'*Escherichia coli* responsables de diarrhées chez le porc en France. *Revue de Médecine Vétérinaire*.

[40] Sidhu J. P. S., Warish A., Hodgers L., Toze S. (2013). Occurrence of virulence genes associated with diarrheagenic pathotypes in Escherichia coliisolates from surface water. *Applied Environmental Microbiology*.

[41] Moyo S. J., Maselle S. Y., Matee M. I., Langeland N., Mylvaganam H. (2007). Identification of diarrheagenic *Escherichia coli* isolated from infants and children in Dar es Salaam, Tanzania. *BMC Infectious Diseases*.

[42] Jacobs S., Holzel A., Wolman B. (1970). Outbreak of infantile gastro-enteritis caused by *Escherichia coli* 0114. *Archives of Disease in Childhood*.

